# Sensitivity Analysis of Geometrical Parameters on the Aerodynamic Performance of Closed-Box Girder Bridges

**DOI:** 10.3390/s18072053

**Published:** 2018-06-27

**Authors:** Yongxin Yang, Rui Zhou, Yaojun Ge, Yanliang Du, Lihai Zhang

**Affiliations:** 1 State Key Lab for Disaster Reduction in Civil Engineering, Tongji University, Shanghai 200092, China; yang_y_x@tongji.edu.cn (Y.Y.); yaojunge@tongji.edu.cn (Y.G.); 2 Institute of Urban Smart Transportation & Safety Maintenance, Shenzheng University, Shenzhen 518060, China; du_yanliang@163.com; 3 Department of Infrastructure Engineering, University of Melbourne, Melbourne, VIC 3010, Australia; lihzhang@unimelb.edu.au

**Keywords:** closed-box girder, wind fairing angle, lower inclined web angle, wind tunnel testing, particle image velocimetry, flutter performance, vortex-induced vibration performance

## Abstract

In this study, the influence of two critical geometrical parameters (i.e., angles of wind fairing, α; and lower inclined web, β) in the aerodynamic performance of closed-box girder bridges was systematically investigated through conducting a theoretical analysis and wind tunnel testing using laser displacement sensors. The results show that, for a particular inclined web angle β, a closed-box girder with a sharper wind fairing angle of α = 50° has better flutter and vortex-induced vibration (VIV) performance than that with α = 60°, while an inclined web angle of β = 14° produces the best VIV performance. In addition, the results from particle image velocimetry (PIV) tests indicate that a wind fairing angle of α = 50° produces a better flutter performance by inducing a single vortex structure and a balanced distribution of the strength of vorticity in both upper and lower parts of the wake region. Furthermore, two-dimensional three-degrees-of-freedom (2D-3DOF) analysis results demonstrate that the absolute values of Part A (with a reference of flutter derivative *A*_2_^*^) and Part D (with a reference of *A*_1_^*^H_3_^*^) generally decrease with the increase of β, while the change of the participation level of heaving degrees of freedom (DOF) in torsion-dominated coupled flutter initially increases, reaches its peak, and then decreases with the increase of β.

## 1. Introduction

Due to its great torsional stiffness, structural stability, and economic advantage, closed-box girders have been widely adopted in long-span cable-supported bridges around the world over the past several decades, such as in the Great Belt Suspension Bridge with a main span of 1624 m and the Russky Island Cable-Stayed Bridge with a main span of 1104 m [1,2,3]. With the ever-growing span length of long-span bridges, the wind-structure interaction resulting from high structural flexibility and low structural stiffness can have a profound impact on the aerodynamic performance of bridges, and thus the functionality and safety of these bridges [4,5]. The most challenging problem encountered by long-span cable-supported bridges with closed-box girders is how to simultaneously improve the critical aerodynamic performance of bridges, especially for their flutter and vortex-induced vibration (VIV) performance. As a self-excited vibration with amplitude divergence, flutter instability—which belongs to the dynamic aeroelastic phenomena and could eventually lead to the failure collapse of the bridge—should be avoided in the wind-resistance design. As an amplitude limiting vibration, vortex-induced vibration (VIV) could result in relatively large-amplitude vibration and discomfort for passengers on the bridges, thus special attention should be also paid to this factor in the design [6,7,8]. Basically, aerodynamic shape is one of important factors for the flutter and VIV performance of long-span suspension bridges, since an excellent aerodynamic shape could not only effectively improve the aerodynamic performance of long-span bridges, but also avoid the high costs in the construction of aerodynamic countermeasures [9,10,11,12]. Thus, it is necessary to further investigate the influence of aerodynamic shape on the flutter and VIV performance of long-span suspension bridges with closed-box girders.

**Figure 1:**
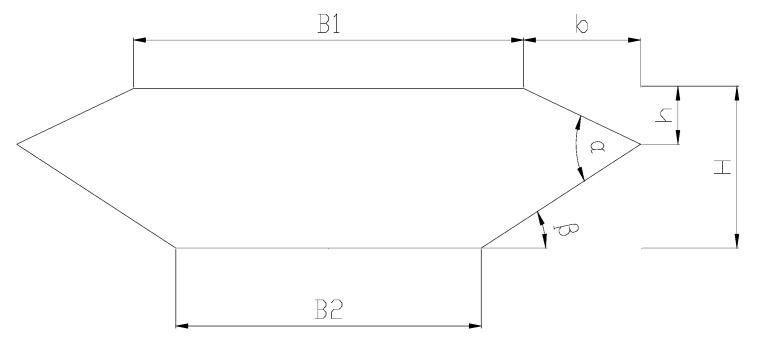
Geometrical parameters of a closed-box girder.

A closed-box girder is formed when two web plates are joined by a common flange at both in the top and the bottom. Figure 1 shows the detailed geometric parameters of a closed-box girder with respect to the aerodynamic performance, such as the depth of the deck (H), widths of the top plate (B1) and bottom plate (B2), angle of wind fairing (α), angle of lower inclined web (β), schemes of aerodynamic appendages, location of the inspection rail, and shape of the guardrail, etc. [13]. The depth and width of the main girder, which are mainly determined by the design requirement of the navigation and traffic levels, cannot be treated as the alterable parameters of the geometrical configuration. It should be mentioned that most of the additional appendages lack versatility and their mechanisms are also not clear, which makes the appendages not easily applied in the sensitivity analysis of aerodynamic shape. Furthermore, the location of the inspection rail and the shape of the guardrail has little influence on flutter instability but are sensitive to the VIV performance of a closed-box girder, which indicates that the high ventilation efficiency of the guardrails and the location of the inspection rail closing to the lower limb of the lower inclined web are more favorable to the aerodynamic performance [14,15]. However, even a minor change in the geometrical configuration of closed-box girders could have great impact on the flow characteristic around the girder and consequently affect the critical aerodynamic performance of bridges. In this study, two important sectional shape-dependent aerodynamic parameters (i.e., the angles of wind fairings (α) and the angles of lower inclined webs (β)) are treated as the primary optimal parameters.

For existing long-span cable-supported bridges with closed-box girders and a main span over 1000 m all over the world, most of the values of α range from 50° to 60° and the values of β range from 10° to 20°, according to the References [13,16]. Based on these facts, two α values of 50° and 60° as the upper and lower boundaries, and six β values from 10° to 20° with intervals of 2° were selected in the current research. Recently, many scholars have studied the effects of wind fairings and lower inclined webs on the aerodynamic characteristics of box girders. Larsen found that a wind fairing with smaller angle has a higher flutter critical velocity though wind tunnel tests [6]. The flap and fairing are often used to improve the aerodynamic stability of the bridge section [13]. Wang reached the conclusion that the flutter critical wind speed increases significantly and the VIV phenomenon disappears when the inclined angle of the lower inclined web turns below 16° for the 4th Nanjing Yangtze River Bridge [17,18]. Larsen and Wall [1] utilized three different bottom plate inclinations and also recommended an angle of around 15° to suppress vortex shedding vibrations. Ito studied the coherence characteristics of fluctuating lift forces for a rectangular shape with various fairing decks and suggested that the upstream fairing tends to weaken the flow separation from the leading edge. Our previous study [19] compared the flutter performance of closed-box bridge decks with five types of geometrical configurations through experimental investigation and found that a sharper angle of wind fairing enhances the flutter performance. We also explored the flutter mechanism by combining of the two-dimensional three-degrees-of-freedom (2D-3DOF) analysis scheme and particle image velocimetry (PIV) tests [20], in which the characteristics of the typical vortices around central-slotted sections which are related to the interactive force between the deck and surrounding airflow vary significantly with the relative slot width. Haque et al. [21] investigated the effect of wind fairing on the aerodynamic stability of the bridge section by using computational fluid dynamics (CFD), and he found that the bottom plate slopes of 25°–15° for top plate slopes of 40° or less have lower aerodynamic responses. He et al. [16] investigated the influences of three typical geometric parameters (i.e., width to height ratio (B1/H), wind fairing angle (α), and wind nose geometry (H/h)) on the aerodynamic forces of a streamlined flat box girder. It showed that the girder with H/h = 2–3 and a relatively larger B1/H and α produces a better aerodynamic performance of the girder. In summary, the previous studies show that a closed-box girder presents excellent aerodynamic performance when the angle of wind fairing becomes sharper and the inclined angle of the lower web is below 16°; however, the relationship between the angle of wind fairing and the inclined angle of the lower web as well as their influence in the aerodynamic performance of bridges are not yet fully understood.

In the present study, the roles of two sectional shape-dependent aerodynamic parameters (i.e., angle of wind fairing (α), angle of lower inclined web (β)) on the critical aerodynamic performance with the consideration of both flutter instability and VIV performance were investigated through a series of wind tunnel tests. In order to establish the relationship between these two parameters of closed-box girders and the flutter performance and VIV performance, the critical flutter wind velocity (U_cr_) of closed-box girders with two different angles of α and six different angles of β were compared, and then the corresponding flutter derivatives, aerodynamic damping of closed-box girders, as well as the flutter performance of some existing long-span cable-supported bridges with closed-box girders were evaluated. Secondly, the velocity field and surface pressure distributions obtained from PIV tests were presented to further understand the flow structures around these closed-box girders with various geometrical configurations. Finally, both the vertical and torsional VIV performance as well as the aerodynamic performance of these bridges were evaluated based on the comparison results of the flutter performance. The sensitivity analysis of two important geometrical parameters of a closed-box girder is helpful to further understand the role of aerodynamic shape on the aerodynamic performance of long-span cable-supported bridges with closed-box girders.

## 2. Experimental Setup of Closed-Box Girders with Various Shapes

**Figure 2:**
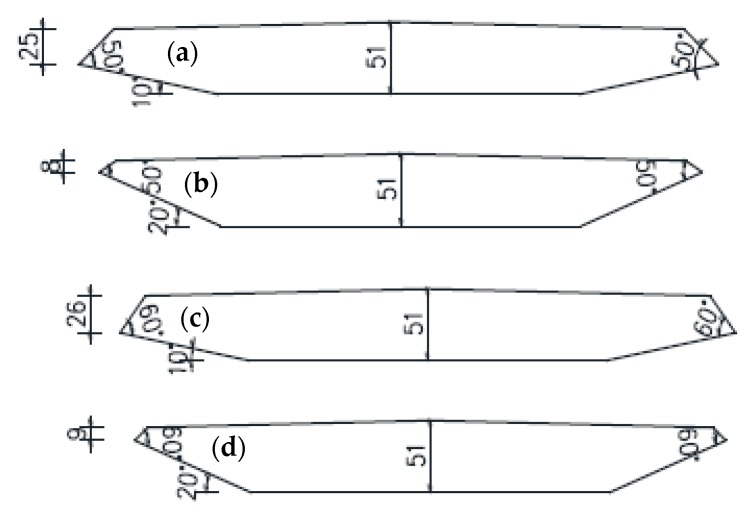
Sectional model diagrams of four closed-box girder sections (unit: cm): (**a**) Section with α = 50° and β = 10°; (**b**) Section with α = 50° and β = 20°; (**c**) Section with α = 60° and β = 10°; (**d**) Section with α = 60° and β = 20°.

**Figure 3:**
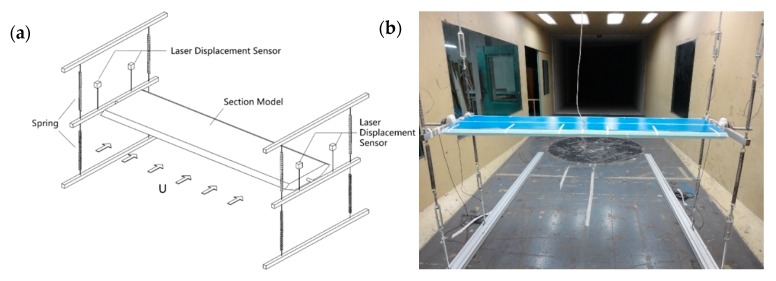
Experimental diagrams for flutter tests and VIV tests: (**a**) Setup; (**b**) Sectional models of flutter tests.

Wind tunnel tests could provide a reliable method to evaluate both the flutter and VIV performance of long-span bridges through laser displacement sensors. To systematically investigate the influence of the wind fairing angle (α) and lower inclined web angle (β) on the flutter and VIV performance of closed-box girders, six representative values of β (i.e., β = 10°, 12°, 14°, 16°, 18°, and 20°) and two extreme values of α (i.e., α = 50° and 60°) were selected in the flutter tests and VIV tests, among which four examples of the closed-box girder sections are shown in Figure 2. The elaborate structural parameters of the sectional models with two α values and six β values are listed in Table 1. It is assumed that all of the damping ratios were 0.5% and the structural dynamic characteristics were consistent for the closed-box girders with the same α. A series of wind tunnel tests on spring-supported two-dimensional rigid sectional models of closed-box girders were conducted in the TJ-1 Boundary Layer Wind Tunnel of Tongji University (Figure 3). The ratio of the geometric scale was 1:70 without the consideration of deck attachments. It is noted that the ratios of the wind speed scale for flutter tests and VIV tests were 1:8 and 1:3, respectively. In total, there were 36 testing cases with three attack angles of +3°, 0°, and −3° for both flutter tests and VIV tests of closed-box girders, as presented in Table 2.

## 3. Flutter Performance Comparison

### 3.1. Comparison of Critical Flutter Wind Speeds

**Figure 4:**
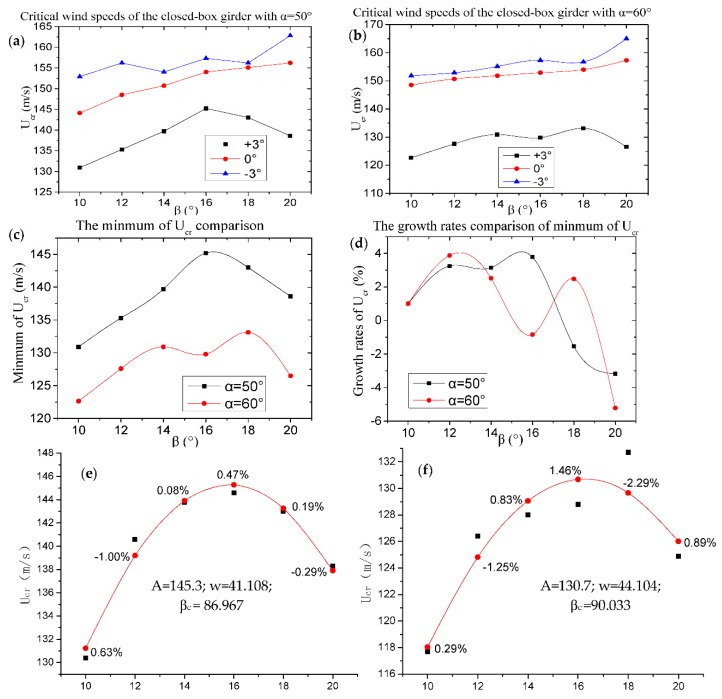
Relationship of U_cr_ and β: (**a**,**b**) Relationship of U_cr_ and β with α = 50° and 60°; (**c**,**d**) Relation of the minimum values and their growth rates of U_cr_ and β°; (**e**,**f**) Fitted function of U_cr_ with α = 50° and 60°.

The relationship between the critical flutter wind speeds (U_cr_) of closed-box girders with two wind fairing angles of α = 50° and 60° are compared in Figure 4. It can be seen that U_cr_ gradually increased with the increase of β under the wind attack angle of +0°, regardless of the wind fairing angle α, and the value of U_cr_ of closed-box girders firstly increased before β = 14° and then decreased after β = 18° under the wind attack angle of +3°. The flutter performance of a girder section is determined by the lowest value of the critical flutter wind speeds among three attack angles, and the largest and lowest critical wind speeds for all of the closed-box girders were the attack values of −3° and +3°, respectively. In particular, the highest value of U_cr_ of the bridge with α = 50° was about 145.2 m/s when β = 16° and the peak of U_cr_ with α = 60° was about 129.8 m/s when β = 18°. As described in Figure 4d, the largest growth rates of the minimum U_cr_ of α = 50° and α = 60° were 3.8% for β = 14° and 3.9% for β = 12°, whereas the smallest growth rates of α = 50° and α = 60° were −3.2% and −5.2% for β = 20°, respectively. It can be concluded that the flutter performance of a relatively sharper β angle of the lower inclined web is superior to that of a relatively blunter β. In addition, all of the values of U_cr_ of closed-box girders with α = 50° are much higher than those with α = 60°, which confirms that closed-box girders with a relatively sharper wind fairing angle have better flutter performances. It is noted that the flutter performance was not obviously improved when the β value was below 16° for the large angle of α = 60°, which is different from the results of the study of Wang et al. (2011) [17].

Besides, the critical flutter wind speeds for closed-box girders with different wind fairing angles and inclined web angles can be fitted using a Sine function, as shown in Equation (1) and Figure 4e,f. It can be seen that the results of a Sine function agree reasonably well with the change in U_cr_ for closed-box girders with various geometrical configurations.

(1) $$U_{\mathrm{cr}}=A\cdot \sin\left[\frac{\pi \left(\beta -\beta_{c}\right)}{w}\right]$$

### 3.2. Comparison of Flutter Derivatives

**Figure 5:**
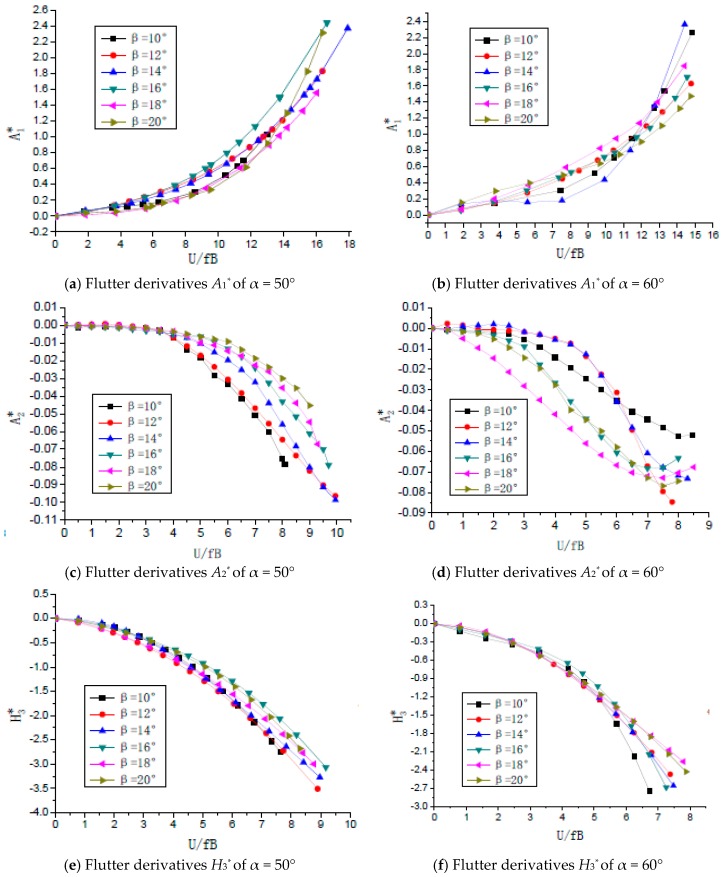
Three important flutter derivatives under +3° wind attack angle: (**a**,**b**) Flutter derivatives *A*_1_^*^ of α = 50° and 60°; (**c**,**d**) Flutter derivatives *A*_2_^*^ of α = 50° and 60°; (**e**,**f**) Flutter derivatives *H*_3_^*^ of α = 50° and 60°.

As shown in Figure 5, the typical flutter derivatives of the closed-box girders with various geometrical configurations were calculated and three important derivatives including *A*_1_^*^, *A*_2_^*^, and *H*_3_^*^ under the most unfavorable wind attack angle of +3° were compared [22]. All of the values of the derivatives *A*_1_^*^ were positive and gradually increased with the increase of the reduced wind speed (U/fB). All of the values of *A*_2_^*^ were negative for all reduced wind speeds and the absolute values of *A*_2_^*^ increased as β increased in the case of α = 50°, which is different from those when α = 60°. In general, the absolute values of *H*_3_^*^ become larger as the reduced wind speed increases or the inclined web angle β decreases, and all values of *H*_3_^*^ were also negative when α = 50° and 60°.

### 3.3. Comparison of Aerodynamic Damping and Flutter Modality

**Figure 6:**
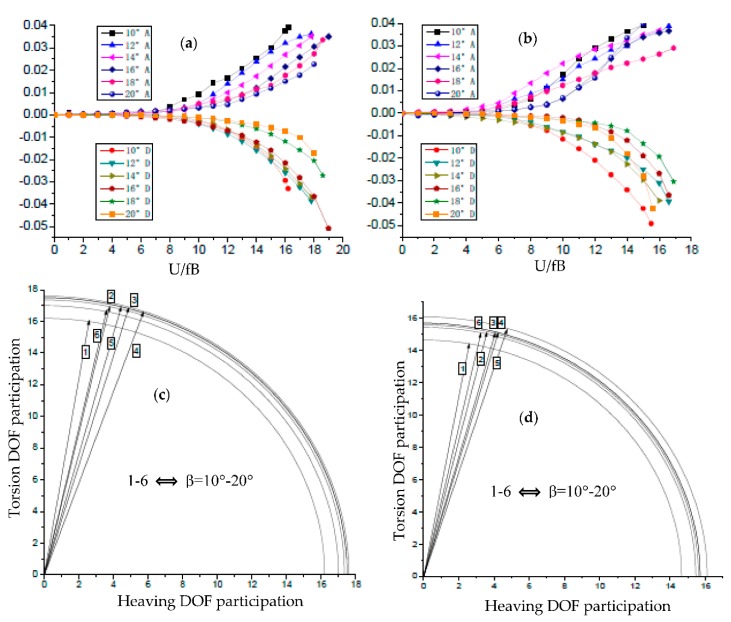
Aerodynamic damping ratios and degrees of freedom (DOF) participation level: (**a**) Aerodynamic damping parts A, D of α = 50°; (**b**) Aerodynamic damping parts A, D of α = 60°; (**c**) Flutter modality vectors of α = 50°; (**d**) Flutter modality vectors of α = 60°.

Based on the 2D-3DOF method [23], two important parts of aerodynamic damping of the closed-box girders with six β values and two α values are plotted in Figure 6a,b, where the expression of aerodynamic damping parts of A and D are introduced in Equation (2), as follows:

(2) $$A=-\frac{1}{2}\frac{\rho B^{4}}{J_{\alpha }}A_{2}^{*};\;D=-\frac{\rho^{2}B^{6}}{2m_{k}I\cdot \;\Omega_{k\alpha }}A_{1}^{*}H_{3}^{*}\cos\theta_{k\alpha }$$

It can be seen that Part A (with a reference of *A*_2_^*^) increased in the positive region with the increase of the reduced wind speed, while Part A significantly decreased with the increase of β when α = 50°. Therefore, Part A makes the greatest contribution to aerodynamic stability. However, Part D (with a reference of *A*_1_^*^H_3_^*^) was negative and decreased with the increase of the reduced wind speed, and so exhibits the worst influence on aerodynamic stability. The absolute values of Parts A and D continued to decrease with the increase of inclined angles of webs. As illustrated in Figure 6c,d, the participation levels of torsion motion of the closed-box girders were considerably high and the distribution of the flutter modality vectors were concentrated. This implies that the coupling flutters are given priority to torsional motions. Moreover, the influence of lower inclined webs in flutter modality can be clearly seen as the participation level of heaving degrees of freedom DOF in the torsion-dominated coupled flutter initially increased, reached its peak, and then decreased with the increase of β. A higher participation level of heaving DOF at the flutter critical condition always leads to a better flutter performance.

### 3.4. Flutter Instability Evaluation of Closed-Box Girders Bridges

The flutter critical wind speed U_cr_ of closed-box girders bridges can be calculated by the following Equation (3) based on the specification of the wind-resistant design for highway bridges [24], where $\eta_{s}$ and $\eta_{\alpha }$ are the girder shape factors with respect to the damping ratio and wind attack angle factor, *f_t_* is the torsional frequency, and *B* is the width of the main girder. This equation shows that U_cr_ is proportional to the structural torsional frequency.

(3) $$U_{\mathrm{cr}}=\eta_{s}\cdot \eta_{\alpha }\cdot U_{\mathrm{cr}}=\eta_{s}\cdot \eta_{\alpha }\cdot \left(2.5\cdot f_{t}\cdot B\cdot \sqrt{\mu \cdot \frac{r}{b}}\right);\mu =\frac{m}{\pi \rho b^{2}};\;\frac{r}{b}=\frac{1}{b}\sqrt{\frac{I_{m}}{m}}$$

In order to evaluate the flutter performance of the closed-box girders with two angles of α and six angles of β, the U_cr_ of some existing bridges with these assumed girder sections were compared with their flutter checking speeds ([U_cr_]), as shown in Table 3. Firstly, the torsional frequencies (*f_t_*) and flutter checking velocities of these practical long-span bridges were surveyed and the ratio of these torsional frequencies of the experimental design model section and practical bridges (λ*_ft_*_=_ 5.429/*f_t_*) were calculated. Secondly, the scaled torsional frequencies ratios λ*_v_* (λ*_v_* = λ*_ft_* × 1/70) of the corresponding sectional model with the same geometric scale and wind speed scale ratio as the sectional model were calculated. Finally, the assumed lowest U_cr_ of these bridges with two angles of wind fairing and six angles of lower inclined web were deduced based on the maximum and minimum values of the lowest flutter critical wind speeds in Figure 5.

As listed in Table 3, three suspension bridges, including the Great Belt Bridge, Runyang Yangtze Bridge, and 4th Nanjing Yangtze River Bridge with the length of the main span ranging from 1418 to 1624 m, were employed for comparison. The values of U_cr_ for α = 50° are higher than those for α = 60° with the same β, and all of U_cr_ for α = 50° meet with the flutter requirement, whereas only the U_cr_ for α = 60° with β = 20° are higher than their respective [U_cr_,]. As for the Jiangyin Yangtze River Bridge whose the main span is below 1400 m, some of the values of U_cr_ for α = 50° are smaller than [U_cr_] = 56.36 m/s while all of the U_cr_ for α = 60° could not meet the flutter requirement. Thus, the experimental girder section with relatively high angles of lower inclined web and sharper angles of wind fairing, is advised to be used in the wind-resistant design. It is should be noted that all of the values of U_crs_ for the long-span cable-stayed Sutong Bridge are greatly inferior to the relative high flutter checking speed of [U_cr_] = 56.36 m/s, while all of the values of U_cr_ of the 3rd Nanjing Yangtze River Bridge with a 648-m main span are much higher than its [U_cr_], regardless of which of the two angles of α are employed.

As a result, some of these long-span bridges with the assumed experimental main girder could exhibit a flutter stability problem, which should be avoided in the wind-resistant design of long-span suspension bridges. Specifically, only the experimental girder section with the sharper angle of α = 50° with the angle of β = 16° could meet the flutter requirement for bridges with a main span below 1400 m, but a sharper angle of α = 50° with all six angles of β could be recommended for suspension bridges with a main span in the range of 1400 to 1624 m in order to avoid the flutter instability. As regards long span cable-stayed bridges, the experimental girder section with two angles of α is sensitive to the flutter performance, as shown through two example analyses of the Sutong Bridge and the 3rd Nanjing Yangtze River Bridge. Therefore, the experimental section with different wind fairings and lower inclined webs angles of a closed-box girder have a certain application range for flutter instability.

## 4. PIV Tests of Closed-Box Girders with Various Shapes

Particle image velocimetry (PIV) enables the accurate, quantitative, and simultaneous measurement of fluid velocity vectors at a very large number of points, and therefore can obtain the related properties of the wind field around the bridge deck [25]. A high-resolution three-dimensional (3D) PIV system including two digital CCD (charge-coupled device) cameras, two laser sheets, a dual Nd:YAG laser, a synchronous controller, and a host computer was used to measure the flow structures around the closed-box girders with various geometrical configurations with respective to the velocity field and vorticity.

### 4.1. 3D PIV System

**Figure 7:**
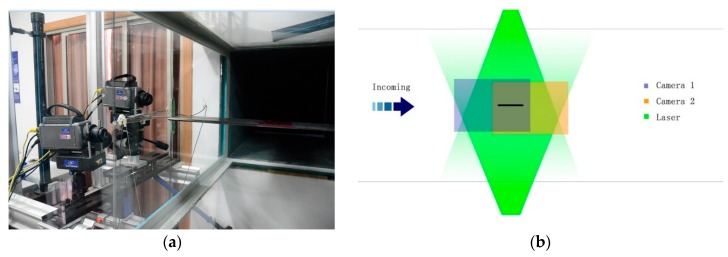
A three-dimensional (3D) particle image velocimetry (PIV) system: (**a**) Experimental setup, and (**b**) Schematic of the 3D flow structures.

In order to reflect the high-resolution three-dimensional flow structures around the bridge deck, two digital CCD cameras and a dual Nd:YAG laser were set outside the working section (which is 0.8 m wide, 0.8 m high, and 5 m long) in Tongji University’s TJ-4 wind tunnel with the maximum wind velocity of 30 m/s, as shown in Figure 7a. Two digital CCD cameras were able to simultaneously capture the region around the sectional models and the slotted parts through the double frame, using the double exposure method, as described in in Figure 7b. The complete flow fields were stitched together by two images of the feasible flow field regions of each camera with the sampling frequency of 200 Hz. The velocity maps were obtained using FFT (Fast Fourier Transform) based on the cross-correlation method in two sequential frames overlapping 50% in each direction with an interrogating window of 64 × 64 pixels. It should be noted that the ratio of the geometric scale is 1:140 and the ratio of the wind speed scale for flutter testing is 1:13.

### 4.2. Comparison of Velocity Fields

**Figure 8:**
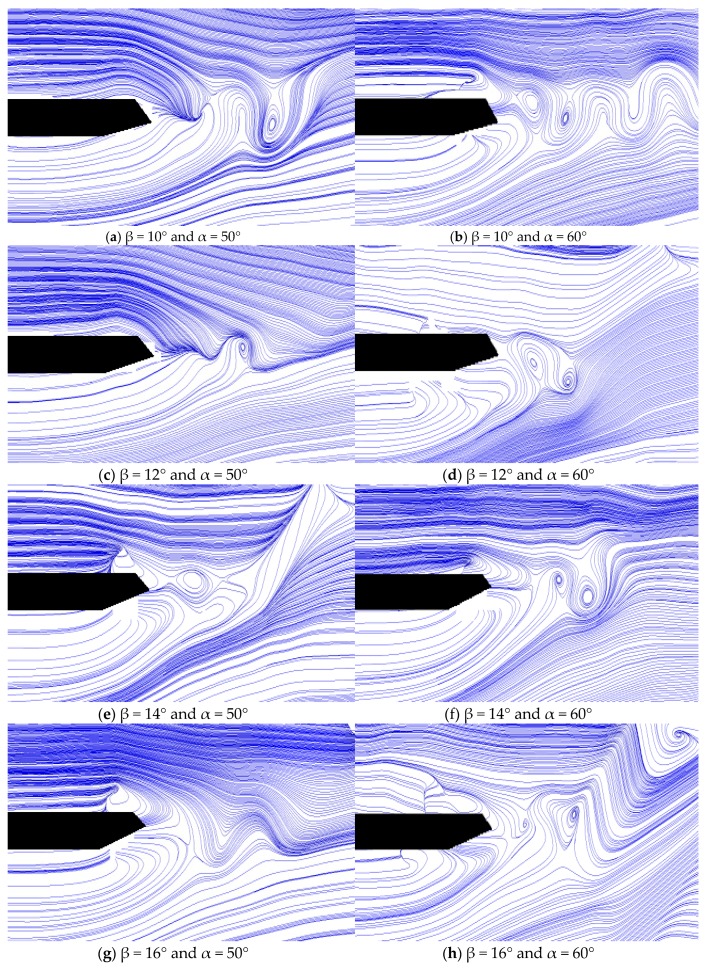

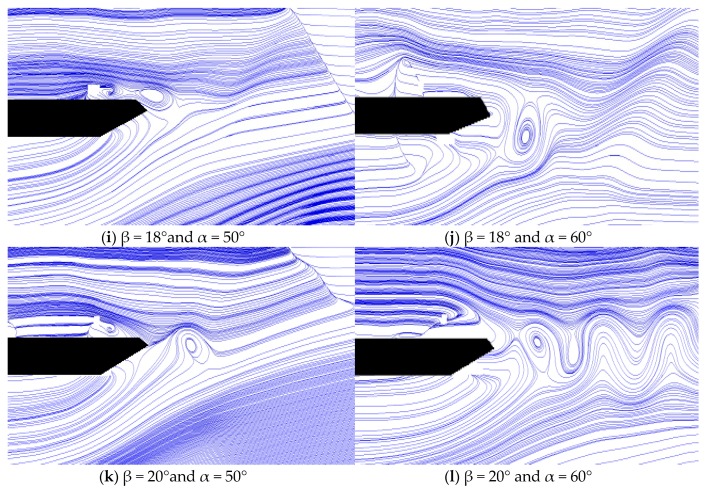
Streamline maps of velocity vector both for α = 50° and α = 60°: (**a**–**l**) β = 10°, 10°, 12°, 14°, 16°, 18°, 20°, respectively.

A series of PIV tests were performed to examine the velocity field of the entire flow field by identifying the motion path and velocity of a tracer in the flow field to further the understanding of the flutter mechanism of closed-box girders with various geometrical configurations. The large eddy simulation decomposition (space-averaged) method was used in the PIV measurement, and the distributions of the ensemble-averaged flow quantities were obtained from a cinema sequence of about 400 frames of instantaneous velocity fields for each test case. The detail velocity vector maps and their streamline maps around the wake regions of these closed-box girders under the wind attack angle of +3° are compared in Figure 8.

Figure 8 shows that the flow structures in the wake regions vary with the wind fairing angle or the lower inclined web angle. For α = 50° in the left section of the maps, a significant single vortex appeared in the wake region for β = 12° and β > 16°. In particular, the small vortexes could be neglected for β = 16°. However, a similar phenomenon did not occur for α = 60° in the right section of the maps. A pair of vortexes were generated in the waking flow β < 16°, which gradually increased with the increase of β. Nevertheless, the vortex structure transformed into a single vortex when β > 16° and gradually decreased with the increase of β. The interaction between the deck and wake flow caused by the movements of vortexes was strengthened when β < 16°. On the other hand, the pair of vortexes for α = 60° could have more significant impact on the participation level of torsional motion in the coupled flutter onset than those of single vortexes for α = 50° with the same value of β. This would lead to a better flutter performance when α = 60°. Therefore, the size of the vortex in the wake region and its interaction with the structural motion could have a significant impact on the participation level of heaving and torsional motion, and therefore contribute to the evolution of the flutter performance of closed-box girders as the geometrical configuration changes.

### 4.3. Comparion of the Characteristics of Vortices

**Figure 9:**
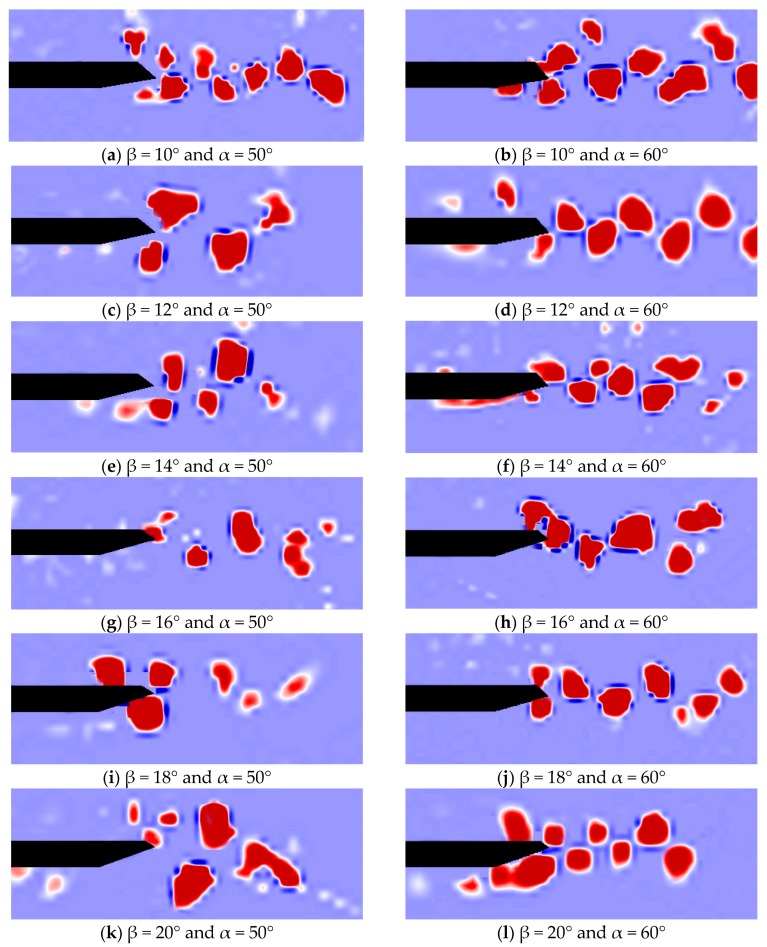
Vortices’ characteristics around the girders both for α = 50° and α = 60°: (**a**–**l**) β = 10°, 12°, 14°, 16°, 18°, 20°, respectively.

As one of the important characteristics of vortices, the vorticity around the wake regions of the closed-box girders with various geometrical configurations were also compared to further investigate the effect of vortex structures on the flutter performance. Figure 9 shows the results of the instantaneous vorticity distributions mainly around the wake regions for the closed-box girders with α = 50° and 60°, respectively. The flow separation of the unsteady vortex first occurred at the edges of the windward wind fairing, then attached to the top and bottom plates, and was eventually shed from the leeward wind fairing. With the increase of β, the dimension of the vorticities in the wake region of the closed-box girders apparently increased when β < 16°, and the number of these vorticities gradually became smaller. This observation was opposite that when β > 16°. The various vortex patterns in the wake regions for different lower inclined web angles could affect the correlation of flow around the bridge deck, which could modify the wind-induced forces and ultimately change the flutter performance. On the other hand, there were high swirling strength vortices distributed both in the upper and lower zones of the wind fairing when α = 50°, while many high swirling strength vortices were concentrated in the lower zones of the wind fairing when α = 60°. More intensive interactions of the vortices when α = 50° were seen than those for α = 60°. Therefore, the wind fairing angle and lower inclined web angle had a great influence on the vorticity and intensive interaction of vortexes in the wake region, which were directly related to the aerodynamic performances of the closed-box girder.

## 5. Comparison of VIV Performance

**Figure 10:**
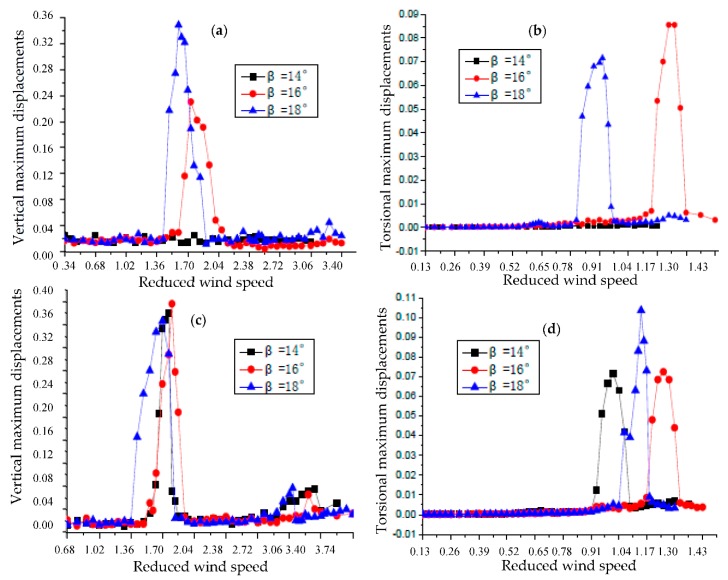
Non-dimensional maximum VIV responses comparison both for α = 50° and α = 60°. (**a**) Vertical maximum VIV displacements for α = 50°; (**b**) Torsional maximum VIV displacements for α = 50°; (**c**) Vertical maximum VIV displacements for α = 60°; (**d**) Torsional maximum VIV displacements for α = 60°.

Since the flutter performance is excellent when the value of β ranges from 14° to 18°, the VIV tests of the closed-box girders with the above three β values and two α values (50°and 60°) were also conducted through laser displacement sensors to identify the optimal VIV performance under the three wind attack angles. The detail lock-in wind speed regions, the non-dimensional maximum amplitudes, and the corresponding wind speeds and Strouhal numbers (St) of the vertical and torsional VIV responses of these bridges are listed in Table 4 (under the wind attack angle of +3°) and Table 5 (under the wind attack angle of 0°). Figure 10 compares the non-dimensional maximum VIV responses under different wind attack angles, in which the attack angle of −3° leads to the smallest VIV responses.

Figure 10 shows that there are no VIV responses of the closed-box girders observed when β = 14° and α = 50°. However, the girders encountered visible heaving and torsional VIV for α = 50° with the largest vertical displacement of 0.017 m at the wind speed of 4.8 m/s when β = 18°, and the largest torsional displacement of 0.086° at the wind speed of 9.6 m/s when β = 16°. As for the larger wind fairing angle of α = 60°, the maximum amplitudes of vertical and torsional VIV responses were 0.02 m at the wind speed of 5.3 m/s when β = 16° and 0.104° at the wind speed of 8.8 m/s when β = 18°, respectively. Although the VIV phenomenon for α = 60° should not be neglected when β = 14°, the amplitudes of vertical and torsional VIV did not reach their maximums under the three β values (i.e., 14°, 16° and 18°). On the other hand, both the vertical and torsional VIV responses of the girders with the angle of α = 50° were smaller than those with the angle of α = 60° and the same value of β, except for the torsional VIV of β = 16°. In summary, the closed-box girder with an angle of β = 14° and a sharper wind fairing angle of α = 50° had the best VIV performance, while the combination of α = 60° with either β = 16° or β = 18° led to the worst VIV performance.

After considering two crucial aerodynamic performances in term of flutter and VIV performance, the closed-box girders with α = 50° and β ranging from 14° to 18° were found to have excellent flutter performance, among which β = 14° had the best VIV performance, followed by β = 18°. As for the girders with α = 60°, β = 14° not only had the best flutter performance but also had the best VIV performance. Therefore, the closed-box girder with the sharper inclined web angle of β = 14° and sharper wind fairing angle of α = 50° should be recommended with consideration of the flutter performance and VIV performance in the current investigation.

## 6. Conclusions

In this study, we systematically investigated two critical aerodynamic performance of closed-box girder bridges (i.e., flutter and VIV performance) with different wind fairing angles and lower inclined web angles through experimental investigation and theoretical analysis. The following are the major findings:

- For a particular inclined web angle β, a closed-box girder with a sharper wind fairing angle of α = 50° has better flutter and VIV performance than that with α = 60°. Among the six inclined web angles of β = 10°, 12°, 14°, 16°, 18°, and 20°, an inclined web angle of β = 14° produces the best VIV performance.
- Based on the 2D-3DOF analysis, the absolute values of Part A (with the reference of flutter derivative *A*_2_^*^) and Part D (with the reference of *A*_1_^*^H_3_^*^) generally decrease with the increase of the lower inclined web angle. In addition, the change of the participation level of heaving DOF in the torsion-dominated coupled flutter initially increases, reaches its peak, and then decreases with the increase of β.
- The results of the PIV tests indicate that the sharper wind fairing angle of α = 50° induces single vortex structures together with a balanced distribution of high strength vorticity in the upper and lower parts of the wake region, consequently producing a better flutter performance than that of α = 60°. Moreover, the inclined web angle β has a great influence on the pattern and intensive interaction of the vortices in the wake region of closed-box girders.

Although the experimental studies in this paper show that the geometrical configuration with β = 14° and α = 50° can produce the best aerodynamic performance, further investigations on the effects of other geometrical configurations of a bridge on the aerodynamic performance of that bridge still need to be carried out to fundamentally understand the underlying mechanisms of wind-induced vibration.

## Figures and Tables

**Table 1:** Parameters of sectional models with different wind fairings and lower inclined webs.

Properties		α = 50°	α = 60°
		β = 10°~20°	β = 10°~20°
Width (B), (m)		0.517~0.548	0.511~0.533
Depth (D), (m)		0.051	0.051
Mass (M), (kg/m)		5.241	5.241
Mass Moment of Inertia (I), (kg·m^2^/m)		0.110	0.110
Damping Ratio, (ζ), (%)		0.5	0.5
Flutter Tests	Vertical frequency (f_v_), (Hz)	2.055	2.055
	Torsional frequency (f_t_), (Hz)	5.429	5.429
Vortex-Induced Vibration (VIV) Tests	Vertical frequency (f_v_), (Hz)	5.480	5.480
	Torsional frequency (f_t_), (Hz)	14.477	14.477

**Table 2:** Testing cases of the closed-box girders sectional models both for flutter and VIV tests.

Case	Angle of Wind Fairing	Angle of Web Plate	Height h (m)	Length L (m)	Width B (m)	Wind Attack Angle (°)
1	α = 50°	10	0.051	1.74	0.548	0, ±3
2		12	0.051	1.74	0.543	0, ±3
3		14	0.051	1.74	0.537	0, ±3
4		16	0.051	1.74	0.531	0, ±3
5		18	0.051	1.74	0.524	0, ±3
6		20	0.051	1.74	0.517	0, ±3
1	α = 60°	10	0.051	1.74	0.533	0, ±3
2		12	0.051	1.74	0.530	0, ±3
3		14	0.051	1.74	0.526	0, ±3
4		16	0.051	1.74	0.521	0, ±3
5		18	0.051	1.74	0.516	0, ±3
6		20	0.051	1.74	0.511	0, ±3

**Table 3:** Assumed flutter performance of some existing bridges with the experimental deck.

Long-Span Bridges	Length of Main Span (m)	Geometrical Parameters		*f_t_* (Hz)	λ*_ft_*	λ_v_	U_crs_ (m/s)		[U_cr_] (m/s)
		Α (°)	β (°)				α = 50°	α = 60°	
Great Belt Bridge	1624	53.1	26.6	0.278	19.529	0.279	58.42~64.78	52.73~59.45	57.96
Runyang Yangtze River Bridge	1490	61.6	16.7	0.225	24.129	0.345	47.25~52.39	42.64~48.08	45.36
4th Nanjing Yangtze River Bridge	1418	50.8	16.0	0.263	20.643	0.295	55.25~61.27	49.87~56.23	51.07
Jiangyin Yangtze River Bridge	1385	52.6	20.1	0.258	21.043	0.301	54.15~60.05	48.88~55.11	56.36
Sutong Bridge	1088	55.4	11.0	0.231	23.502	0.336	48.51~53.79	43.79~49.37	60.14
3rd Nanjing Yangtze River Bridge	648	54.9	16.6	0.621	8.749	0.125	130.39~144.58	117.71~132.71	41.99

**Table 4:** Non-dimensional VIV response comparison under the wind attack angle of +3°.

VIV Responses	β (°)	α = 50°				α = 60°			
		Lock-In Wind Speed Region (m/s)	Maximum Amplitude (m/°)	Peaking Wind Speed (m/s)	St	Lock-In Wind Speed Region (m/s)	Maximum Amplitude (m/°)	Peaking Wind Speed (m/s)	St
Vertical	14	/	/	/	/	1.56–1.80	0.035	1.77	0.108
	16	1.62–2.03	0.023	1.75	0.102	1.65–1.96	0.038	1.82	0.106
	18	1.46–1.95	0.032	1.67	0.107	1.46–1.88	0.036	1.74	0.109
Torsional	14	/	/	/	/	0.85–1.00	0.068	0.95	0.161
	16	0.87–1.03	0.077	0.95	0.144	0.88–1.01	0.067	0.96	0.161
	18	0.82–0.98	0.072	0.94	0.150	0.87–1.05	0.040	0.92	0.170

**Table 5:** Non-dimensional VIV response comparison under the wind attack angle of 0°.

VIV Responses	β (°)	α = 50°				α = 60°			
		Lock-In Wind Speed Region (m/s)	Maximum Amplitude (m/°)	Peaking Wind Speed (m/s)	St	Lock-In Wind Speed Region (m/s)	Maximum Amplitude (m/°)	Peaking Wind Speed (m/s)	St
Vertical	14	/	/	/	/	/	/	/	/
	16	/	/	/	/	/	/	/	/
	18	/	/	/	/	/	/	/	/
Torsional	14	/	/	/	/	0.90–1.08	0.072	1.00	0.153
	16	1.14–1.30	0.086	1.25	0.114	1.17–1.33	0.073	1.25	0.124
	18	/	/	/	/	1.05–1.20	0.104	1.16	0.135
